# Analysis of 5-Azacytidine Resistance Models Reveals a Set of Targetable Pathways

**DOI:** 10.3390/cells11020223

**Published:** 2022-01-11

**Authors:** Lubomír Minařík, Kristýna Pimková, Juraj Kokavec, Adéla Schaffartziková, Fréderic Vellieux, Vojtěch Kulvait, Lenka Daumová, Nina Dusilková, Anna Jonášová, Karina Savvulidi Vargová, Petra Králová Viziová, Radislav Sedláček, Zuzana Zemanová, Tomáš Stopka

**Affiliations:** 1 BIOCEV, 1st Medical Faculty, Charles University, 25250 Vestec, Czech Republic; Lubomir.Minarik@vfn.cz (L.M.); Kristyna.Pimkova@lf1.cuni.cz (K.P.); juraj.kokavec@lf1.cuni.cz (J.K.); adela.schaffartzikova@email.cz (A.S.); fvell@lf1.cuni.cz (F.V.); kulvait@gmail.com (V.K.); lenka.daumova@gmail.com (L.D.); nina.dusilkova@lf1.cuni.cz (N.D.); anna.jonasova@vfn.cz (A.J.); 2 Clinic Haematology, General Faculty Hospital, 12808 Prague, Czech Republic; 3 Pathophysiology, 1st Medical Faculty, Charles University, 12853 Prague, Czech Republic; karin.vargova@lf1.cuni.cz; 4 Czech Centre for Phenogenomics, Institute of Molecular Genetics, 25250 Vestec, Czech Republic; petra.kralova-viziova@img.cas.cz (P.K.V.); radislav.sedlacek@img.cas.cz (R.S.); 5 Cytogenetics, General Faculty Hospital, 12808 Prague, Czech Republic; zuzana.zemanova@vfn.cz

**Keywords:** myelodysplastic syndrome, Azacytidine, resistance, CDX mice, PI3K/AKT signaling

## Abstract

The mechanisms by which myelodysplastic syndrome (MDS) cells resist the effects of hypomethylating agents (HMA) are currently the subject of intensive research. A better understanding of mechanisms by which the MDS cell becomes to tolerate HMA and progresses to acute myeloid leukemia (AML) requires the development of new cellular models. From MDS/AML cell lines we developed a model of 5-azacytidine (AZA) resistance whose stability was validated by a transplantation approach into immunocompromised mice. When investigating mRNA expression and DNA variants of the AZA resistant phenotype we observed deregulation of several cancer-related pathways including the phosphatidylinosito-3 kinase signaling. We have further shown that these pathways can be modulated by specific inhibitors that, while blocking the proliferation of AZA resistant cells, are unable to increase their sensitivity to AZA. Our data reveal a set of molecular mechanisms that can be targeted to expand therapeutic options during progression on AZA therapy.

## 1. Introduction

Patients with MDS suffer from a clonally evolving hematologic malignancy with altered myeloid differentiation and cytopenias, which often progresses to AML. HMA therapy with AZA has been established for higher-risk MDS (n = 358) [1] and elderly AML (n = 113) [2] patients providing longer overall survival (OS) and improved therapeutic response compared to the conventional chemotherapy. However, the response duration usually lasts less than 1-year in both MDS [3] and AML [4]. Other MDS/Myeloproliferative neoplasms (MPN) include Chronic Myelomonocytic Leukemia (CMML), where AZA therapy is beneficial, as shown in a study of 48 patients with an overall response rate of up to 70% (according to the International working group (IWG) criteria), including 22% complete responses [5]. Upon loss of responsiveness to HMA the patient survival markedly shortens [6]. Simple decitabine replacement after AZA treatment failure in patients with MDS and CMML is clearly not an option, as shown in a study in which the median survival from decitabine initiation was 7.3 months, with no significant difference between responders and non-responders [7]. Several studies indicated that specific DNA variants associate with a negative impact on overall survival largely due to HMA resistant disease [8] and predict the development of HMA resistance [9]. However, understanding of the molecular changes during the development of HMA failure remains incomplete, hindering the development of new therapeutic agents.

In order to better understand the mechanisms of resistance to HMA, we have to describe how HMA works towards MDS. Conservation of epigenetic repression in every cell is induced de novo or perpetuated upon replication by enzymes catalyzing the addition of a methyl group to the 5′ carbon of the cytosine ring resulting in 5-methylcytosine. Cytosine-rich regions are often located at regulatory loci near genes and thus DNA methylation is critical for gene expression pattern formation and consequently a cell phenotype. AZA and also decitabine are analogs of the nucleoside cytidine that are incorporated into DNA during replication forming thus 5-aza-dCTP, which is recognized and irreversibly bound by maintenance DNA methylase DNMT1 [10]. Inhibition of DNA methylation has become an established therapeutic approach in MDS, although the mechanism of action remains only partially elucidated at the molecular level, primarily involving a defective DNA methylation pattern and the presence of variants in genes involved in this process (e.g., *DNMT3A*, *TET2*, *IDH1/2*). The efficacy of HMA can be affected by enzymatic activities of HMA uptake and activation although their clinical relevance remains also unclear (see references in [11]).

There already exists growing evidence of therapies affecting pathways involved in HMA resistance. For example, the combination of HMA and the BCL2 inhibitor Venetoclax (VEN) is effective towards patient subgroups including secondary cases as well as bearers of *Tumor Protein P53* (*TP53*) mutation [12]. However, *TP53* inactivation may play an important role in the efficacy of HMA, but data from a study with a p53 activator molecule (APR-246) together with AZA are not yet available. Another study with Rigosertib for MDS patients during the HMA failure was designed to target RAS and phosphatidylinosito-3 kinase (PI3K) pathways, however, the mechanism of action is not well understood [13]. Another study investigates the efficacy of a combination of AZA and Pevonedistat, a NEDD8-activating enzyme inhibitor [14]. The addition of a drug that either counteracts AZA resistance or is able to induce leukemia cell toxicity by targeting the mechanisms that AZA resistant cells use to survive is likely to prolong OS. AZA resistant cells can tolerate a number of drugs but relatively little is known about which drugs have significant efficacy. In general, the lower the concentration of a drug that can be used to inhibit the growth of AZA resistant cells, the lower the predicted side effects of the therapeutic will be.

To identify target pathways in AZA resistant cells, we first established a model from the MDS/AML cell line that was primarily sensitive to AZA. We also used primary MDS samples from AZA-treated patients who had progressed during this treatment. From these systems, we identified a spectrum of DNA variants and differentially expressed programs at the mRNA level. By integrating these data, we identified AZA resistance pathways and tested whether inhibitors blocking these pathways could inhibit the cell growth and, if so, whether they could affect AZA sensitivity.

## 2. Materials and Methods

Outline of the study: Azacytidine resistant and control MDS/AML subclones were generated and subject to in vivo validation in the immunocompromised mice. Next generation sequencing and cytogenetics were used to identify the mutation and expression profiles. Selected specific inhibitors were used to test the AZA resistant mechanisms.

Generation of AZA resistant clones: We used AZA sensitive OCI-M2 (AZA-S), which was originally derived from a 56-year-old MDS-EB2 patient in transition to AML (DSMZ collection, #ACC 619). Two other MDS/AML lines were also AZA-S: MOLM-13 (DSMZ, #ACC 554, derived from a 20-year-old male with MDS-EB in relapse to MDS/AML); SKM1 (DSMZ, #ACC 547, derived from a transition to MDS/AML). AZA resistant (AZA-R) subclones were obtained using a similar procedure as shown by a previous study [15]. Initially, cells were cultured for 48 h at a concentration of 10.000/well in triplicate 96-well plates in Iscove’s Modified Dulbecco’s medium (IMDM) + 20% fetal bovine serum (FBS) +1% Penicillin/Streptomycin (150 μL media/well). Subsequently, AZA was added at concentrations of 0.1, 1, 5, 8, 10 μM with the agent being replenished every 2 days. The medium was changed every week. Individually growing clones were obtained, propagated, and subsequently, frozen aliquots were stored. The WST1 is a cell proliferation colorimetric assay (Roche, Basel, Switzerland) to obtain IC50 (concentration of drug required for 50% inhibition).

Cell Line Derived Xenograft (CDX) mice are triple transgenic non-obese diabetic—Scid gamma mice expressing human Interleukin-3, Granulocyte Macrophage-Colony Stimulation Factor and Stem Cell Factor that combine the features of the highly immunodeficient mice producing the cytokines to support engraftment of myeloid lineages (NSG-SGM3, NSGS). Transplantation into NSGS mice was intraosseous (femoral bone) [16] and with both AZA-S and AZA-R cells bearing Luciferase expression to weekly monitor tumor cell expansion under general anesthesia with isoflurane. Extracted tumors (at the end of the experiment for each mouse) were also confirmed by flow cytometry to express human-specific surface antigen CD45. D-luciferin in 100 μL was injected intraperitoneally (i.p.) prior to luminescence detection in the warming plate in SPECTRAL Lago X Imaging System. Data for the calculation of radiance were obtained from Spectral Instruments Imaging by Aura Imaging Software. Radiance is a calibrated absolute measurement of photon emission from the subject (photons/second/cm^2^/steradian). Mean Rad is defined as Total radiance/number of pixels in the ROI (region of interest) defined as the image area quantified. Tumor growth was monitored once a week as established elsewhere [17]; AZA therapy in mice consisted of 12 i.p. injections of AZA per month at a dose of 150 μg Vidaza (Celgene) per mouse. The dose was very similar to that for MDS patients i.e., 7 × 75 mg/m^2^ per month.

Whole exome sequencing (WES) utilized DNA monoplicates from AZA-S cells, AZA-R subclones (#1, #20, #33), and DNAs from the MDS sample biobank. We used the Roche NimbleGen SeqCap Kit (Roche, Indianapolis, IN, USA) covering 47 Mb of all medically relevant genes on the Illumina NextSeq 500 platform (Illumina, San Diego, CA, USA) [18], achieving a median depth of coverage of 50×. NGS data were analyzed using standard quality assay tools (FastQC, FastQ screen, multiQC), mapping to the genome (GRCh38) with alignment (BWA MEM) and removal of redundant sequences to generate SAM/BAM files. The mapped data as VCF files were annotated through databases (dbSNP, ClinVar). We focused on data with read depth (>10) and variant allele frequency (>5%). Only variants with high (score 4/5) and very high impact (score 5/5) on protein structure, among others, frameshift, stop-gain, or structural changes were considered.

Multicolor fluorescence in situ hybridization (mFISH). Chromosomal spreads were conducted according to standard cytogenetic methods using colcemid, hypotonic treatment, and fixation in methanol/acetic acid. Karyotypes were analyzed with the mFISH method using the 24 XCyte color kit and an ISIS computer analysis system (MetaSystems, Altlussheim, Germany) according to manufactures’ protocols. Where possible, at least 10–20 metaphases were karyotyped for each sample and the findings were described according to cytogenetic nomenclature.

RNA sequencing (RNAseq) of AZA-S cells and AZA-R subclones either untreated or incubated with 1 µM AZA for 24 h was used as established previously [19]. mRNA was magnetically isolated using Poly(A) mRNA Magnetic Isolation Module (NEBNext, New England Biolabs, Ipswich, MA, USA) from 1 µg of total RNA after residual DNA depletion using DNA-free Kit (Thermo Fisher Scientific, Waltham, MA, USA). Paired-end libraries were prepared using the NEBNext Ultra II Directional RNA Library Kit with NEBNext multiplexed index oligonucleotides for Illumina (Dual Index Primers Set 1). Biological duplicates were used, thus for each mentioned sample, we created two separate libraries. Libraries were sequenced on an Illumina NextSeq500 instrument with paired-end reads of 150 bp per sample. Sequence data were aligned with the STAR aligner program (2.7.3a) to the human reference genome GRCh38.p13 (Ensembl 103). Differentially expressed genes (out of 24,855 mRNAs) were identified using DESeq2 as those whose differential expression was greater than 2-fold (up/down) with a Benjamini-Hochberg corrected *p*-value. The PANTHER classification system was used to obtain gene classification [20].

Annotation: Gene lists of DNA variants, differentially expressed mRNAs or combined list, respectively, were submitted to DAVID Functional annotation clustering tool. The DAVID Functional annotation clustering function uses a Kappa statistic score to measure relationships among the gene ontology (GO) annotation terms. Enrichment analysis was used to highlight the most relevant GO terms associated with given gene lists [21]. GO terms with a *p*-value < 0.05 were considered as significantly enriched [22]. GAD, KEGG, GOTERM, BIOKARTA and INTERPRO analyses were performed. GraphPad Prism and R were used for data visualization.

## 3. Results

### 3.1. Development of a Cellular Model for AZA Resistance

**Figure 1:**
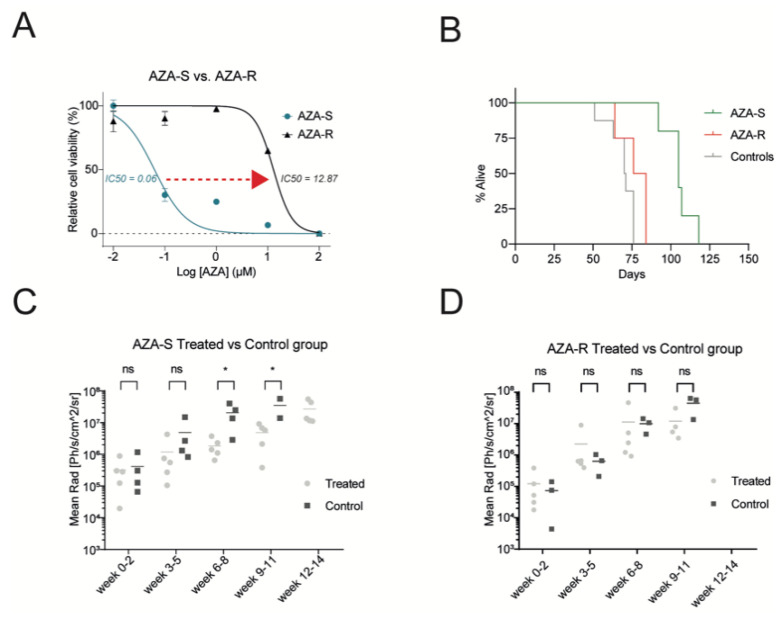
Analysis of the AZA resistance model. (**A**) WST1 assay of AZA-sensitive (S) vs. AZA-resistant (R) cells (clone #1); IC50^AZA^ is indicated. (**B**–**D**) AZA-S & AZA-R cells were transplanted into NSGS mice and treated with AZA or vehicle (Control). Therapy of 150 μg AZA/mouse was applied i.p. three times weekly. Survival was monitored (days indicated, 4 or 5 mice per each group). AZA-S vs. AZA-R *p* = 0.004, AZA-S v Ctrl *p* = 0.0014, AZA-R vs. Ctrl *p* = 0.0953. (**C**,**D**) Luciferase detection (*y*-axis Mean Rad) in control vs. AZA-treated mice bearing AZA-S (left) or AZA-R (right) xenograft cells. Analysis utilized unpaired Mann–Whitney *t*-test. * *p*-value < 0.05, ns not significant.

We used cell lines (OCI-M2, SKM1, and MOLM-13) that represent the progression of MDS to MDS/AML to generate AZA resistant subclones by escalating in vitro concentration of AZA. AZA-R subclones from OCI-M2 cells achieved an optimal level of IC50 (Figure 1A, Supplementary Figure S1A) demonstrating their AZA resistance. The proliferation rate was comparable between AZA-R and AZA-S cells, albeit slightly slower in AZA-R cells (Figure S1B). We engrafted AZA-R or AZA-S cell lines in the bone marrow of immunocompromised NSGS mice and tested their sensitivity to AZA. As expected, we observed proliferation and tumor formation of AZA-S and AZA-R subclones, which were composed of cells with typical features of myeloblasts with fine chromatin and bulky nuclei (Figure S1C). Treatment of CDX mice with AZA resulted in prolonged survival in the AZA-S model, whereas AZA-R and control CDX mice had significantly shorter survival (Figure 1B and Figure S1D). Tumors visualized by luciferase detection in AZA-S mice were effectively inhibited by AZA therapy (Figure 1C and Figure S1D), whereas no effect of AZA was observed in the AZA-R model (Figure 1D and Figure S1D). Thus, AZA-R CDX lines carry a stable AZA resistance phenotype, and we studied them further.

### 3.2. Identification of Pathogenic AZA-R DNA Variants

Mutations in some MDS-associated genes predict the therapeutic efficacy of HMA (e.g., *TET2*, *DNMT3A*, *IDH1*, *IDH2*, *ASXL1*, *CBL*, *RAS*, and *SF3B1* [23]). Using sequencing of a panel of 33 MDS-related genes [24] we detected variants in some of them (*ASXL1*, *BCORL1*, *TP53*, *TET2*, *CSF3R*, *NRAS*) in the AZA-R subclones, none of which, however, arose newly (Figure S1E). We also used cytogenetic tools (mFISH, Figure S1F) and compared AZA-S and AZA-R subclones but did not detect any newly acquired and persistently recurrent specific chromosomal aberrations, so we focused on identifying point variants at the genome level. We, therefore, used WES and identified a total of 537 highly pathogenic variants, 188 of which were shared (Figure S1G left) while 349 were specific to AZA-R and not found in AZA-S (Figure S1G right), 19 of which were detected across AZA-R clones (Figure S1H). Data from WES crossvalidated the data obtained from sequencing of a panel of 33 MDS-related genes. To enrich our data for a more clinically relevant situation than the in vitro system, we used paired MDS samples from patients before and after the development of AZA resistance (Figure S1I) and identified an additional set of AZA-R-specific variants (n = 410). The total number of pathogenic AZA-R DNA variants from in vitro cultures and patients was 947. (with 15 variants overlapping between the two systems). We focused only on a subset of unique AZA-R variants (n = 482, corresponding to 451 Entrez IDs). We observed that among the genes carrying unique AZA-R mutations were genes previously associated with cancer progression (*AKT1*, *HDAC6*, *HDAC2*, *MARCKS*, *SMARCA2*, *BRCA2*, *FANCD2*, *FANC1*, *ERCC1*). To gain an overall insight into the AZA-R mutanome, we used the DAVID resource and studied the association of a set of genes with their biological function, structure, and their involvement in different biological pathways [21]. AZA-R mutanome (Figure S1J,K) associated with tumorigenesis with preferential involvement of several signaling pathways related to the regulation of cell proliferation and survival, especially the calcium and PI3K/AKT signaling pathways. Mutanome data were also related to several key nuclear processes, such as telomere length maintenance, chromatid cohesion, DNA repair, and transcriptional repression. To further explore these results, we needed to determine whether the mutated molecular pathways in AZA-R cells are also affected at the level of mRNA.

### 3.3. Identification of Molecular Patterns for AZA Resistance

**Figure 2:**
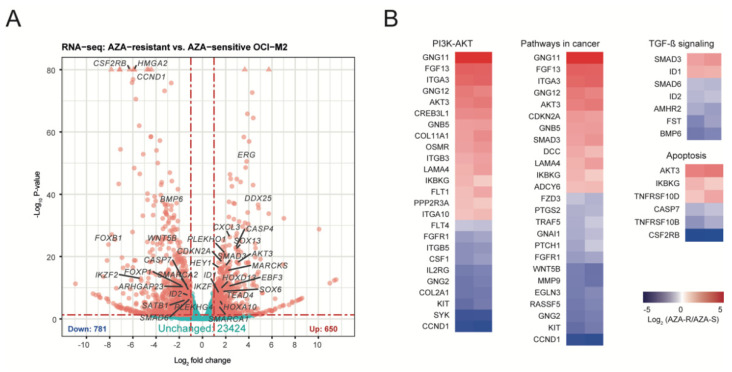
Transcriptomic analysis of cells either resistant or sensitive to AZA. (**A**) Volcano plot of differential gene expression; significance indicated by adjusted *p* < 0.05; log2 fold change expression > 1 is marked by red. Selected mRNAs are displayed with HGNC symbol. (**B**) Heatmaps show mRNA expression log2(AZA-R/AZA-S) in two replicates as revealed by KEGG pathway analysis.

To identify acquired programs of AZA resistance we performed a global analysis of mRNA expression using RNAseq technology. A similar approach previously associated the WNT pathway with primary AZA resistance [25]. The transcriptomic analysis using RNAsec revealed 2865 differentially expressed mRNAs between AZA-S and AZA-R (adjusted *p* < 0.05; mRNAs with at least 1-fold down/up-regulation are shown in Figure 2A), which were enriched for several key biological processes related to apoptosis, chemokine signaling, PI3K/AKT, RAP1, TNF, TGF, and cancer (Figure 2B and Figure S2A). While AZA-S cells responded to AZA treatment by differentially expressing 939 genes, AZA-R cells altered the expression of 191 genes upon AZA treatment, confirming that their sensitivity to AZA is significantly lower (data not shown). We focused on those mRNAs whose mRNA expression differed significantly (and at least twofold) between AZA-S and AZA-R cells. The AZA-R set contained a total of 602 differentially expressed (Entrez ID: n = 563) mRNAs, whereas 298 mRNAs were upregulated and 304 mRNAs were downregulated in AZA-R cells. Again, we used the DAVID resource to obtain an overall overview of the AZA-R pattern [21].

By annotating the mRNA profiles of AZA-R (Figure S2B,C), we again observed strong associations with cancer, but also with metabolism and hematology (GAD_disease) programs. KEGG analysis revealed that AZA-R profiles are linked to phosphorylation regulation of specific signaling pathways, such as chemokine, PI3K/AKT, TGFβ, and RAS pathways that interfere with cell cycle regulation and survival also through regulating GTPase activity (GOTERM_BP, GOTERM_MF). INTERPRO analysis revealed the involvement of the Pleckstrin domain that is known to mediate intracellular signaling via phosphatidylinositol. Based on a combined approach, i.e., analysis of overlapping AZA-R outcomes from WES and RNAseq (Table 1, Figure S2D), we further extended the spectrum of disrupted specific pathways and confirmed the role of PI3K/AKT, chemokine, RAP1, and RAS signaling in the AZA-R phenotype. In addition, we also noted strong associations of GTPase and protein kinase/phosphatase activities and roles of Pleckstrin, Src Homology 2, and protein kinase domains. Our next step was to validate these data using selected drugs that directly or indirectly modulate the aforementioned AZA-R dysregulated pathways.

### 3.4. Dysregulation of the PI3K-AKT Pathway in AZA-R Cells

**Figure 3:**
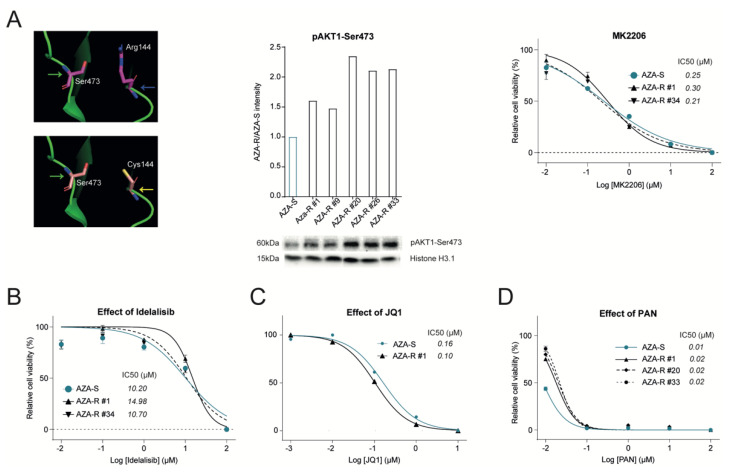
Validation of AZA resistance pathways. (**A** *left*) structural model of *AKT1* variant c.430C>T (p.Arg144Cys), unmutated Arg (top, blue) vs. mutated Cys (bottom, yellow), phosphorylated Ser473 in orange; (**A** *middle*) AKT1-Ser473 phosphorylation (Western blotting, densitometry on top); (**A** *right*) WST1 assay using AKT1 inhibitor (MK2206, AZA-S vs. AZA-R). (**B**) WST1 assay; Idelalisib (IDE); (**C**) BET inhibitor JQ1; (**D**) HDAC inhibitor Panabinostat (PAN); AZA-R clones indicated by #.

Among the AZA-R variants with a significant overlap between subclones and with a significant allelic frequency that we identified with WES is the *AKT1* c.430C>T mutation (p.Arg144Cys). We modeled how *AKT1* c.430C>T (p.Arg144Cys) may function (Figure 3A). Based on the 3D structure, the major signaling residue Ser^473^ that is phosphorylated via the upstream PI3K-mediated actions becomes stabilized by the neighboring side chain of Arg^144^ that forms a salt bridge with it. The accessibility of the Ser^473^ side-chain would, therefore, be reduced by the presence of the Arg^144^ side chain. However, the presence of the arginine side chain is seen as a stabilizing factor once phosphorylation has taken place.

The R144C mutation provides a much shorter side chain, and therefore, makes the Ser^473^ side chain more accessible for phosphorylation by the intervening kinase, as can be understood from modeling studies (using the Coot software): the side chain of Arg^144^ was mutated to Cys^144^ (Figure 3A) to show that the Cys side chain may be able to form a stabilizing interaction with the Serine hydroxyl group or the phosphate group of P-Ser^473^. It should be noted that the pK of the Cysteine side chain (~8.3), while lower than that of Arginine (~13.5), retains a mild basic character. The model predicts that R144C is a gain of function (GOF) mutation positively regulating the amount of phosphorylation of the Ser^473^. We confirmed increased phosphorylation in all tested AZA-R subclones (Figure 3A). AKT1 mutation was detected in all AZA-R subclones with high frequency (Figure S1H). AKT1 inhibition using MK2206 was very efficient (IC50~0.3 μM) in AZA-R cells and reached the efficacy observed in the AZA-S cells (Figure 3A). Interestingly, both AZA-S and AZA-R cells were resistant to PI3K inhibition (upstream of AKT1) (IC50~10.2 vs. 12.8 μM) by kinase inhibitor Idelalisib (Figure 3B), which targets the PI3K p110 isoform δ with high potency and selectivity. In addition, the RNAseq data also revealed upregulation of AKT2, AKT3 and MTOR in AZA-R cells confirming the PI3K/AKT pathway overactivation. Autonomous hyperphosphorylation and activation of AKT1 can have a number of downstream effects including the mTOR activation. We, therefore, tested the sensitivity of the AZA-R model to the mTOR inhibitor Rapamycin, which proved to be extremely potent, confirming the importance of AKT1 pathway hyperactivation in this AZA-R cell model.

### 3.5. Validation of Other Signaling Pathways Deregulated in AZA-R Cells

Next, we tested the roles of additional tyrosine kinases implicated in the AZA-R phenotype using a drug screening approach. Dasatinib (DAS) is an oral dual inhibitor of BCR/ABL and SRC family tyrosine kinases, which are non-receptor protein tyrosine kinases involved in the control of a number of cellular processes including proliferation, differentiation, motility, and adhesion. DAS has a mild, consistent and very similar effect on the inhibition of AZA-S and AZA-R cells in the WST1 assay (IC50 ~8 μM; Figure S3A). Another kinase inhibitor, Sorafenib (SOR), acts by inhibiting several different kinases (RAF-1, VEGF, c-KIT, PDGFR, ERK, and FLT3) involved in tumor cell proliferation and angiogenesis. SOR inhibits proliferation by blocking the kinase activity of C-RAF/B-RAF as well as MEK and ERK phosphorylation. However, SOR has a mild effect on the inhibition of AZA-S (IC50 4 μM) and AZA-R cells (IC50 8 μM) in the WST1 assay (Figure S3A). Another inhibitor, Ruxolitinib (RUX) works by inhibiting the signaling of cytokines and their receptors, which use the kinase machinery of JAK1 or JAK2 proteins for signaling that involves transcription activators (STATs) that transduce signals to the nucleus to induce gene transcription. The JAK/STAT pathway is more activated in some AZA-R clones, as seen by the detection of phosphorylated STAT3 on Tyr705 (Figure S3B), an indicator of JAK/STAT signaling activity. However, compared to DAS or SOR, RUX has a milder effect on the inhibition of AZA-S and AZA-R cells in the WST1 assay (IC50 13 vs. 15 μM; Figure S3A). Our data show that blocking a particular kinase activity in AZA-R with DAS, SOR or RUX is relatively ineffective.

As indicated by RNAseq data, dysregulated expression of *ERG*, *FLI1*, *HOXA9*, *HOXA10* suggests the involvement of chromatin regulator bromodomain-containing protein 4 (BRD4). It was recently shown that inhibition of BRD4 suppresses leukemia progression [26]. Therefore, we used JQ1, an inhibitor of BET-domain proteins that are known to be enriched at super-enhancers, which are putative oncogenic drivers required for the maintenance of cancer cell identity. Using WST1 assay we noted that JQ1 had a considerable effect (IC50~0.1 μM) on AZA-R as well as on AZA-S cells (Figure 3C). Data from WES that identified several Histone Deacetylase variants of *HDAC2* and *HDAC6* coupled with dysregulation of HDAC9 led us to test the efficacy of HDAC inhibition in AZA-R cells. We used Panabinostat (PAN), which is a non-selective inhibitor of class I (HDAC 1, 2, 3, 8), class II (HDAC 4, 5, 6, 7, 9, 10) and class IV (HDAC 11) HDACs. The IC50 for PAN was four orders of magnitude lower compared to AZA in AZA-R subclones whereas PAN was similarly effective as AZA in AZA-S cells (IC50~0.01 μM, Figure 3D). Thus blocking BET domains and HDACs appears to be very effective towards AZA-R cells in vitro.

The RNAseq data also indicated that the relationship between cell survival and apoptosis (Figure 2B) induction may play a role in AZA resistance. We focused on BCL2 homologs and their influence on the cell survival pathway and used specific inhibitors including VEN, ABT-737 and S63845. VEN inhibits BCL2 protein, which leads to programmed cell death of malignant stem cells. ABT-737 is known to inhibit BCL-2, BCL-XL and BCL-W but not MCL1 dependently on the presence of BAK or BAX. S63845 is a selective MCL1 inhibitor. Since BCL2, MCL1, and BCL-XL, can partly compensate for each other, we quantified them and show that AZA-R cells up-regulate (2–2.5×) MCL1 while expression of BCL-XL and BCL2 was down-regulated (Figure S3B). The differential cellular expression of BCL2, MCL1 and BCL-xL proteins in AZA-R vs. AZA-S cells may be related to differences in the therapeutic efficacy of these inhibitors. Thus, in monotherapy, VEN, ABT-737 and S63845 have quite differential effects towards AZA-R with S63845 (IC50~0.1–0.4 μM) being the strongest AZA-R inhibitor and VEN being the weakest (IC50~10–14 μM) (Figure S3A).

### 3.6. Addressing Effect of Selected Signaling Pathway Inhibitors on AZA Resistance

Next, we investigated whether selected inhibitors of signaling pathways that are in AZA-R cells quite efficient may modulate AZA resistance. We used again proliferation assay and determined the efficacy of an inhibitor in the presence or absence of AZA. It is important to note here that the added constant concentration of AZA was chosen to have no effect on the growth of AZA-R cells in terms of inhibiting proliferation, but at the same time to be able to fully inhibit the growth of AZA-S cells. The experiment was performed at 1 μM AZA (Figure S3C) and also at 2 μM (not shown) in two AZA-R subclones. Additive or synergistic effect was evaluated in the following inhibitors: MK2206, IDE, DAS, SOR, RUX, JQ1, PAN, VEN, and MCL inhibitor S63845 (Figure S3C). Our data consistently confirm that while some of these inhibitors effectively block the growth of AZA-R cells, they are unable to enhance the effect of AZA in any way. Our data suggest that since the AZA-R phenotype is unaffected by inhibitors of the signaling pathways tested, which are clearly dysregulated, AZA resistance appears to be a conserved state and thus, the cells have lost the ability to return to the state from which they evolved upon long-term incubation with AZA. Thus, this is probably a phenomenon that appears to be difficult to influence, but which we are studying further.

## 4. Discussion

Our goal was to create an in vitro model of MDS/AML cells that upon long-term AZA exposure develop AZA-R phenotype and using this model, but also using primary samples, we have identified dysregulated pathways in order to target them. An important outcome of this work is the involvement of the PI3K/AKT pathway in the AZA-R phenotype. Activation of PI3K is under normal conditions initiated outside the differentiating cells upon exposure to hormones and cytokines via receptor tyrosine kinase, which recruits the adaptor-subunit p85 of PI3K, which is activated by G protein-coupled receptors or small Ras GTPase that bind PI3K directly. The set of phosphorylated intermediates (PIP2, PIP3, PDK1) is graduated by phosphorylation at Ser473 by the mTOR complex, which is thought to be an indicator of activation of this pathway. This pathway was significantly implicated in AZA-R from both WES (Figure S1J,K) and RNAseq (Figure 2B and Figure S2B,C) analyses, and was the overall top ranked pathway in the combined analysis (Table 1). We noted some interesting features of the *AKT1* variant (as suggested by the structural analyses), which is a gain-of-function (GOF)-type mutation c.430C>T (p.Arg144Cys) that leads to hyperphosphorylation and which was also confirmed in AZA-R subclones (Figure 3A). In addition, RNAseq data also revealed upregulation of AKT2, AKT3 and mTOR in AZA-R cells, suggesting excessive activation of the PI3K/AKT pathway (Figure S2B,C). Using specific inhibitors, we subsequently showed that blocking AKT or mTOR, but not PI3K (Figure 3A,B), can inhibit AZA-R cell survival. These data are consistent with a number of previous indications in the literature and follow the extensive work on PI3K/AKT/mTOR in AML, whereas there are not so many new reports on PI3K/AKT/mTOR in MDS progressing on AZA therapy. For example, the evaluated response of 80 samples of primary AML samples to selective inhibitors of the PI3K/AKT/mTOR confirmed that ~60% of them display the pathway hyperactivation, which possibly also segregates with shortened OS [27]. Hyperactivation of the PI3K/AKT/mTOR pathway, including consistent detection of hyperphosphorylation of AKT-Ser473, which we also confirmed in AZA-R subclones, can be typically found in patients with chemoresistant AML, likely related to a mechanism that allows leukemic stem cells to survive [28]. Interestingly, the activation of the PI3K/AKT/mTOR pathway is also related to other pathways in AML, namely *FLT3-ITD* mutations or mechanisms involving BCL2 and the cell survival and apoptosis pathway, which implies that the use of PI3K/AKT/mTOR inhibitors in monotherapy is severely limited. Furthermore, there is a connection to another signaling pathway RAS/RAF/MEK/ERK through mutual regulation and effector sharing, whereby these pathways influence a number of key cellular functions ([29]). Although MK2206 effectively suppresses AKT phosphorylation, it has not been successful in monotherapy (similar to PI3Ki), but its good tolerability opens the possibility of its inclusion in combination strategies for AZA-R MDS. This is supported by data claiming AKT-Ser473 is hyperphosphorylated in 90% of high-risk MDS patients in comparison to low-risk MDS patients or normal controls. Similarly, PI3K p110δ isoform level followed the same pattern of association in myeloblasts of progressing MDS patients [30]. Another paper recently showed that *ASXL1* mutations, which are often associated with lower PTEN levels, exhibit significantly higher sensitivity to the AKT inhibitor MK2206, consistent with the fact that excessive PI3K/AKT/mTOR activation may be a direct consequence of HMAs targeting the general mechanism of transcriptional repression (hence protection of chromatin structure) as well as AML cell survival [31].

Other signaling pathways studied in AZA-R cells include RAS/RAF/MEK/ERK and SRC. The RAS/RAF/MEK/ERK pathway, which consists of a kinase cascade that is regulated by phosphorylation and dephosphorylation by specific kinases, phosphatases, as well as GTP/GDP exchange and other proteins, has been previously published as an indicator of sensitivity and resistance to antileukemic therapy. The key is the phosphorylation of ERK1(MAPK3) on Thr202/Tyr204, which is significantly downregulated in all AZA-R subclones (Figure S3B), and therefore, the kinase inhibitor SOR, which blocks RAF kinase proteins and phosphorylation of MEK and ERK, has relatively little effect on AZA-R cells. This signaling pathway is often dysregulated in resistant cells to various antileukemic agents [32]. However, the use of SOR in AML patients is quite effective, especially in patients with FLT3-ITD mutation [33], leading to prolonged survival [31], again reinforcing the importance of the RAS/RAF/MEK/ERK signaling pathway. Regarding signaling through a family of different Src kinases (including LYN and SRC), these are expressed in AML and their phosphorylation regulates leukemia cell proliferation and survival (including through LYN mediating mTOR activation) [34]. SOR or DAS monotherapy against AZA-R was not effective at all (note that SRC-Tyr416 phosphorylation was not different when comparing AZA-R with AZA-S, see Figure S3B), nor was SOR/AZA or DAS/AZA synergism observed (Figure S3C).

We also studied some nuclear processes associated with AZA-R. WES suggested the involvement of the Cohesin complex and transcription regulation, which was supported by in silico analysis showing the involvement of chromatin remodeling enzymes (Figure S1J). We also observed a role for HDAC and a set of genes *ERG*, *HOXA10* and *KIT* suggesting the involvement of super-enhancers that are >20 kb with an affinity for transcriptional coactivators from the BRD4 family of BET proteins that recognize different histone modifications. Indeed, AZA-R cells are highly sensitive to PAN or JQ1, which inhibits MYC through BRD4 suppression (Figure 3C,D). This is supported by in vivo studies in patients with high IPSS-R risk MDS, CMML, or AML, all unsuitable for transplantation, in whom the PAN + AZA combination doubled the composite remission rate compared to the AZA arm; however, overall response rate and 1-year survival rates were similar in both arms [35], so further research in this direction is needed. Similar to PAN, inhibition of BRD4 in vitro by JQ1 (but also by *BRD4* shRNA) led to apoptosis of a panel of leukemia lines, and this effect was simultaneously enhanced in the presence of AZA [26]. Furthermore, BET inhibitors were recently demonstrated to be highly effective in vivo using AML xenograft models [36]. These results linked the BRD4-dependent transcriptional program to the pathogenesis of MDS and AML and further supported cooperation with the effect of HMA. It is likely that disruption of histone acetylation (by PAN) or inhibition of BRD4 and thus of super-enhancers (by JQ1) may lead to other effects at the chromatin level of AZA-R cells, for example, affecting SWI/SNF helicases (Figure S1J) or affect other nuclear processes related to DNA methylation [37].

## 5. Conclusions

Our work provides new insights into the mechanisms of AZA resistance phenotype and targetable pathways. Specifically, we observe that the AZA-R phenotype involves among several pathways the PI3K/AKT signaling. We anticipate that the use of cellular models and paired samples of MDS patients who have developed resistance may help to find targetable pathways that can be further explored in mouse models in vivo in order to find new therapeutic strategies for AZA resistance.

## Figures and Tables

**Table 1:** DAVID annotation of combined variants and expression sets of AZA resistance involving categories including disease association (GAD), bio-pathways (KEGG), GO terms (biological process, BP) and (molecular function, MF), keywords (upregulated, UP), protein domains (INTERPRO).

Tool	Category (Count ^1^)	*p*-Value ^2^	Genes ^3^
GAD_dis.	cancer (232)	9 × 10^−6^	*AKT1*, *AKT3*, *IKZF1*, *IKZF2*, *IKZF3*, *ERG*, *KIT*, *SMAD3*, *CCND1*, *CDKN2A*, *RUNX1*
	metabolic (335)	3 × 10^−3^	*KIT*, *KLF13*, *KLF6*, *PBX3*, *SMAD3*, *SMAD6*, *SOX13*, *CDH2*, *CTH*, *DPP10*, *EVI5*, *FGFR1*, *FLT1*
	hematologic (239)	4 × 10^−3^	*ERG*, *SATB1*, *SMARCA2*, *CDKN2A*, *AKT3*, *ERCC1*, *JMJD1C*, *SYK*, *HGF*
UP_KEY	protein phosphorylation (534)	6 × 10^−21^	*AKT1*, *AKT3*, *BCOR*, *BMX*, *ACVR1*, *FLT1*, *FLT4*, *FOXP1*, *FOXL2*, *PTPN6*, *PTPN13*, *ERG*
	alternative splicing (629)	2 × 10^−16^	*ARL13B*, *EEF1A1*, *GFM1*, *PABPC1*, *PARG*, *PARP14*, *PARP3*, *PARP8*, *PARP9*, *PCBP2*
KEGG	PI3K-AKT signaling (38)	5 × 10^−5^	*AKT1*, *AKT3*, *CCND1*, *CSF1*, *PTK2*, *PIK3R3*, *FLT1*, *FLT4*
	chemokine signaling (24)	2 × 10^−4^	*CCR7*, *CXCL16*, *CXCL2*, *CXCL3*, *SHC1*, *PLCB3*, *VAV1*, *VAV2*
	Rap1 signaling (25)	5 × 10^−4^	*CSF1*, *EGFR*, *FGF13*, *ID1*, *SKAP1*, *RGS14*, *HGF*
	pathways in cancer (37)	1 × 10^−3^	*BRCA2*, *KIT*, *SMAD3*, *CDKN2A*, *EGFR*, *FZD3*, *HDAC2*, *RUNX1*
	Ras signaling pathway (25)	3 × 10^−3^	*ARF6*, *GNG11*, *GNG12*, *GNB5*, *GNG2*, *RASSF5*, *SHC1*, *KSR1*
GO_BP	GTPase activity (57)	4 × 10^−6^	*AGAP1*, *ASAP2*, *ASAP3*, *GDI1*, *RAP1GAP2*, *RALGPS2*, *ARHGAP23*, *RGS12*, *RGS14*, *RGS20*, *TRIO*
	regulation of proliferation (23)	3 × 10^−4^	*BMX*, *TAL1*, *CDCA7*, *ERBB3*, *HOXD13*, *PTK2B*, *PTK2*, *KIT*
	protein kinase activity (10)	7 × 10^−4^	*CCR7*, *RASSF2*, *CSF1*, *PTPRC*
	signal transduction (86)	1 × 10^−3^	*AKT1*, *AKT3*, *BMX*, *ERG*, *RIN3*, *SHC1*, *IL9R*, *ERBB3*, *CSF2RB*, *MOK*, *KIT*
GO_MF	protein binding (525)	4 × 10^−6^	*ABCA1*, *BCOR*, *DNA2*, *EHD2*, *KLF6*, *SATB1*, *ACVR1*, *CCND1*, *CDKN2A*, *HOXA10*, *HDAC2*, *HDAC6*, *HGF*, *HMGA2*
	GTPase activity (33)	2 × 10^−5^	*AGAP1*, *ASAP2*, *ASAP3*, *CDC42EP1*, *DLC1*, *GDI1*, *RAP1GAP2*, *ARHGEF6*, *RIN3*, *RINL*, *RGS12*, *RGS14*, *RGS20*
	phospholipid binding (14)	5 × 10^−4^	*SHC1*, *DAPP1*, *STAP1*, *AGAP1*
	protein kinase binding (36)	7 × 10^−4^	*BCL10*, *CYLD*, *SMAD3*, *CCND1*, *CDKN2A*, *HCLS1*, *MAPK6*, *PTK2*, *PTPN6*, *PTPRC*, *PTPRK*, *SYK*, *SKAP1*
INTERPRO	Pleckstrin-like domain (53)	2 × 10^−9^	*AKT1*, *AKT3*, *AGAP1*, *BMX*, *RALGPS2*, *SHC1*, *PLEKHA4*, *PLEKHA6*, *PLEKHO1*, *PTK2B*, *PTK2*, *STAP1*
	Pleckstrin domain (37)	8 × 10^−8^	*VAV1*, *VAV2*, *TRIO*, *PLEKHG4*, *PLCL1*, *GRB14*, *DOK4*, *CDH2*, *ARHGEF6*, *ASAP2*, *ASAP3*
	Src Homology 2 domain (18)	4 × 10^−5^	*BMX*, *RIN3*, *RINL*, *SLA*, *GRB14*, *PTPN6*, *SYK*
	Ser-Thre/Tyr- kinase (19)	2 × 10^−4^	*KIT*, *EGFR*, *FLT1*, *FLT4*, *KSR1*, *MAP3K7*, *PTK2B*, *PTK*, *ROR1*, *TIE1*

^1^ minimum gene counts of an annotation term ^2^ Bonferroni Šidák *p*-value ^3^ Name/Gene ID.

## Data Availability

Data supporting reported results can be found at the Array express. RNAseq accession#: E-MTAB-10635, WES data accession#: E-MTAB-11172.

## Supplementary Materials

The following supporting information can be downloaded at: https://www.mdpi.com/article/10.3390/cells11020223/s1, Figure S1: Validation and Mutation screen of AZA-R model & MDS patients. Figure S2: RNAseq and DAVID annotations of AZA-R profiles. Figure S3: WST1 assay of AZA-R inhibitors in monotherapy and with AZA; Immunoblotting analysis for candidate pathways.
