# Supplementary material for: Analysis of 5-Azacytidine Resistance Models Reveals a Set of Targetable Pathways
Source: Cells. 2022 Jan 11;11(2):223. doi: 10.3390/cells11020223 (PMC8774143; doi:10.3390/cells11020223)
Supplement: Supplementary file 1 [file cells-11-00223-s001.zip › cells-1533897-final SM.pdf]

Cell line	AZA-S ($IC50^{AZA}$ µM)	AZA-R ($IC50^{AZA}$ µM)
OCI-M2	0.06	2.52-12.87
MOLM-13	0.30	2.36
SKM1	2.67	5.94

(**A**)

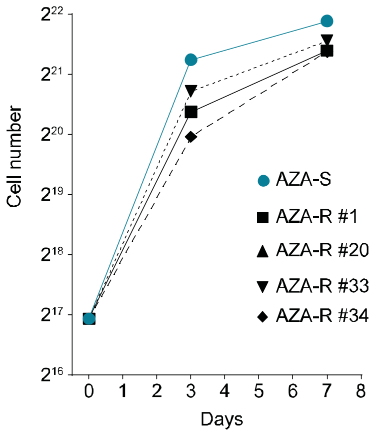

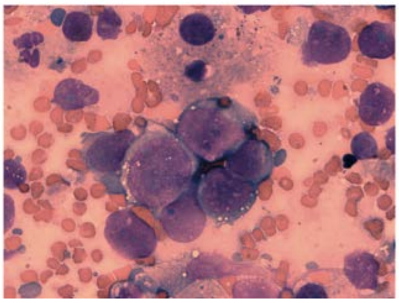

(**B**) (**C**)

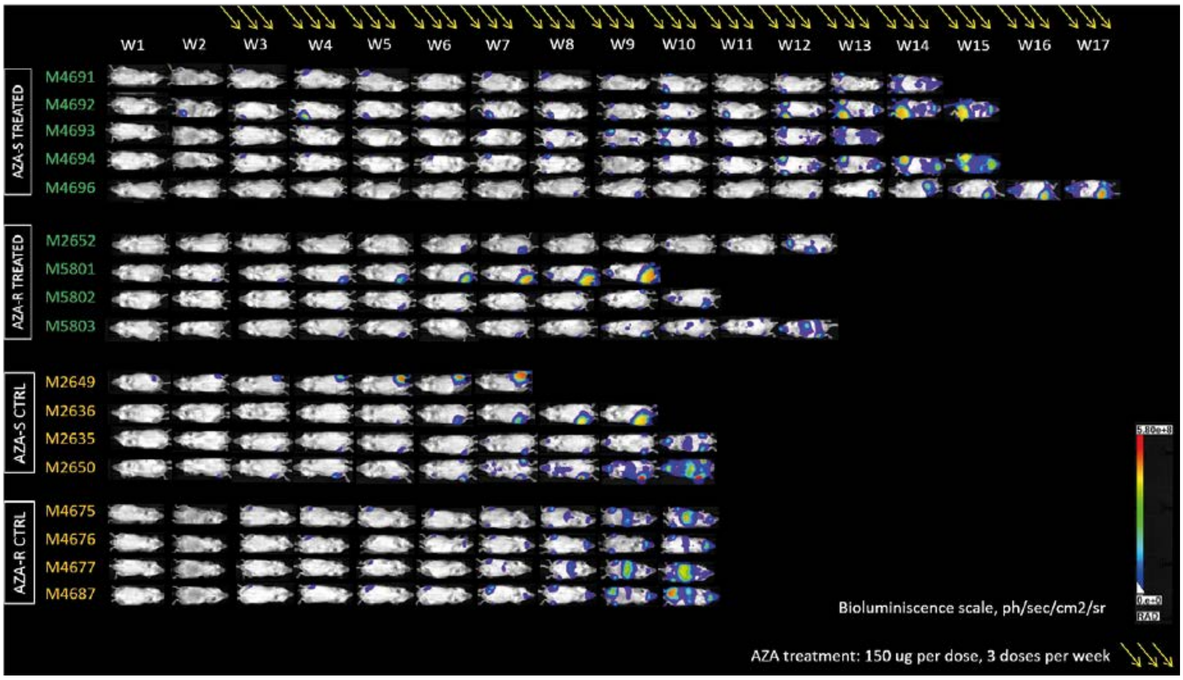

(**D**)

Gene	CDS mutation	AA mutation	Impact	AZA-S	AZA-R #1	AZA-R #20	AZA-R #33
BCORL1	c.331T>C	p.Phe111Leu	missense_variant	100%	100%	100%	100%
ASXL1	c.2444T>C	p.Leu815Pro	missense_variant	100%	100%	100%	100%
TP53	c.215C>G	p.Pro72Arg	missense_variant	99%	100%	-	-
TP53	c.821T>A	p.Val274Asp	structural_interaction_variant	99%	100%	100%	99%
TET2	c.3251A>C	p.Gln1084Pro	missense_variant	50%	51%	46%	46%
CSF3R	c.2092C>T	p.Arg698Cys	missense_variant	49%	68%	-	67%
NRAS	c.181C>A	p.Gln61Lys	missense_variant	46%	44%	46%	54%
ASXL1	c.2128G>C	p.Gly710Arg	missense_variant	41%	-	-	-

(**E**)

Lineage	Karyotype
OCI-M2 AZA-S	45~53,X,der(X)t(X;8)(q13;?),+6,+7,del(7)(p12),+8,der(9)t(9;19)(p23;?)ins(19;21)(?;?),der(10)t(10;12)(p11.1;p13),der(12)t(10;12)(p11.1;p11.2),+14,+14, del(17)(q11q21.1),+20,der(21)t(19;21)*[cp9]<br>*Comment: unstable –set of non-clonal chromosome aberrations*
OCI-M2 AZA-R #1	49,X,der(X)t(X;8)(q13;?),+6,+7,del(7)(p12),+dic(8;14)(p11.1;p11.1),der(9)t(9;19)(p23;?)ins(19;21)(?;?),der(10)t(10;12)(p11.1;p13),der(12)t(10;12)(p11.1;p11.2), del(17)(q11q21.1),der(19)t(1;19)(p?13;?p11),der(21)t(19;21)t(16;19)****[cp9]<br>49,idem,der(16)t(6;16)(?;p13.3)[cp3]<br>*Comment: relatively stable – two clones and other mitosis with non-clonal chromosome aberrations, 4x polyploidy*
CDX	51,X,der(X)t(X;8)(q13;?),+2,dup(2)(q?),+6,+8,der(9)t(9;19)(p23;?)ins(19;21)(?;?),der(10)t(10;12)(p11.1;p13),der(12)t(10;12)(p11.1;p11.2),der(13)t(13;15)(q34;?q26),+14, del(17)(q11q21.1),+20,der(21)t(19;21)*[10]<br>50,X,der(X)t(X;7)(p11.2;?)t(X;8)(q21;?),+6,+8,der(9)t(9;19)(p23;?)ins(19;21)(?;?),der(10)t(10;12)(p11.1;p13),der(12)t(10;12)(p11.1;p11.2),+14,del(17)(q11q21.1),+20,der(21)t(19;21)*[2]<br>*Comment: relatively stable – two clones and one mitosis with non-clonal chromosome aberrations, 1x polyploidy*

AZA-S

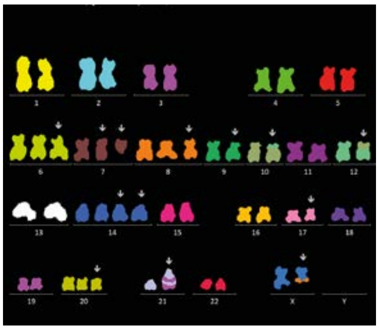

AZA-R, #1

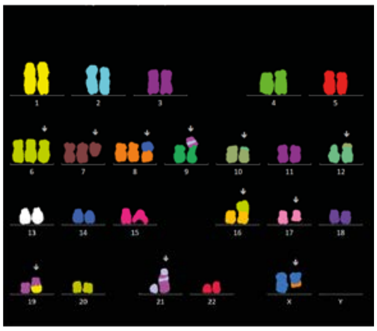

AZA-R, CDX

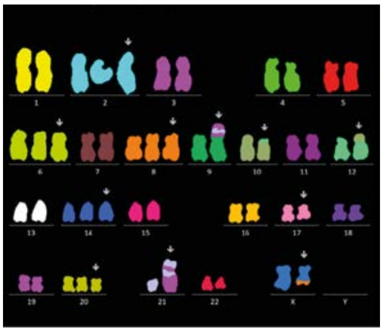

(F)

TYPE	EFFECT	IMPACT	GENE
SNV	stop_gained	HIGH	CFHR3
Insertion	frameshift_variant	HIGH	OR2T35
Insertion	frameshift_variant	HIGH	PRDM15
SNV	structural_interaction_variant	HIGH	NONO
SNV	structural_interaction_variant	HIGH	ARSB
SNV	structural_interaction_variant	HIGH	TP53
Deletion	frameshift_variant	HIGH	PKD1L3
Deletion	structural_interaction_variant	HIGH	POLRMT
SNV	start_lost	HIGH	OTOR
SNV	start_lost	HIGH	TSR3
SNV	structural_interaction_variant	HIGH	AKR1C2
SNV	structural_interaction_variant	HIGH	CTH
SNV	structural_interaction_variant	HIGH	TPSAB1
SNV	structural_interaction_variant	HIGH	ACOT4
Deletion	frameshift_variant	HIGH	TMEM25
SNV	structural_interaction_variant	HIGH	IL12B
Deletion	frameshift_variant	HIGH	CA14
SNV	splice_donor_variant&intron_variant	HIGH	MCPH1
SNV	splice_acceptor_variant&intron_variant	HIGH	EXO1
SNV	splice_acceptor_variant&intron_variant	HIGH	PLA2G4D
Insertion	frameshift_variant	HIGH	MTHFD2
SNV	stop_gained	HIGH	CNTROB
SNV	splice_acceptor_variant&intron_variant	HIGH	KIAA0196
Insertion	frameshift_variant&stop_gained	HIGH	UXS1
Complex	structural_interaction_variant	HIGH	ACOT4
Complex	stop_gained	HIGH	DRAP1
Insertion	frameshift_variant	HIGH	CEP162
SNV	stop_gained	HIGH	HABP2
Deletion	frameshift_variant	HIGH	HCN2
SNV	structural_interaction_variant	HIGH	ANGPT2
SNV	structural_interaction_variant	HIGH	FLT3

TYPE	EFFECT	IMPACT	GENE
SNV	splice_donor_variant&intron_variant	HIGH	FAM227A
Insertion	frameshift_variant	HIGH	KRT4
SNV	structural_interaction_variant	HIGH	DDX3X
Insertion	frameshift_variant	HIGH	TAOK3
SNV	structural_interaction_variant	HIGH	ERCC1
SNV	stop_gained	HIGH	FLG
SNV	stop_gained	HIGH	IKZF3
SNV	stop_gained	HIGH	FAT1
SNV	stop_gained	HIGH	KRTAP1-1
SNV	structural_interaction_variant	HIGH	AKT1
SNV	protein_protein_contact	HIGH	PRIM2
Insertion	frameshift_variant	HIGH	ZFHX3
Insertion	frameshift_variant	HIGH	TBC1D10B
SNV	stop_gained&splice_region_variant	HIGH	BCOR
Deletion	frameshift_variant	HIGH	EEF1A1
SNV	stop_gained	HIGH	PABPC3
SNV	structural_interaction_variant	HIGH	MYOM1
Deletion	frameshift_variant	HIGH	SRD5A3
SNV	structural_interaction_variant	HIGH	SEC23A
SNV	stop_gained	HIGH	SERGEF
SNV	splice_acceptor_variant&intron_variant	HIGH	DGAT1
Insertion	frameshift_variant	HIGH	EGR1
Insertion	frameshift_variant	HIGH	CARF
SNV	structural_interaction_variant	HIGH	EGFR
SNV	stop_gained	HIGH	GTF2H1
SNV	protein_protein_contact	HIGH	DHH
SNV	structural_interaction_variant	HIGH	RAB11A
Deletion	frameshift_variant	HIGH	CCDC63
SNV	stop_gained	HIGH	MYBPHL
SNV	stop_gained	HIGH	BTN2A1
SNV	structural_interaction_variant	HIGH	ROR2
SNV	structural_interaction_variant	HIGH	MEP1B
SNV	protein_protein_contact	HIGH	CTNNB1
SNV	structural_interaction_variant	HIGH	AP4M1
SNV	structural_interaction_variant	HIGH	TFRC
SNV	splice_donor_variant&intron_variant	HIGH	QTRT1
SNV	stop_lost	HIGH	ZNF442
SNV	structural_interaction_variant	HIGH	AIFM1
SNV	stop_gained	HIGH	CXCR3

(G)

Gene	CDS mutation	AA mutation	Impact	AZA-S	AZA-R #1	AZA-R #20	AZA-R #33
FLG	c.6442G>T	p.Gly2148*	stop gained	0%	43%	46%	44%
ASTE1	c.1969delA	p.Arg657fs	frameshift variant	0%	14%	16%	11%
SRD5A3	c.825_829delAACTT	p.Thr276fs	frameshift variant	0%	18%	18%	14%
SLC9B1	c.913C>T	p.Arg305*	stop gained	0%	26%	31%	23%
FAT1	c.13128C>A	p.Cys4376*	stop gained	0%	42%	49%	33%
PRIM2	c.586C>G	n/a	protein protein contact	0%	21%	41%	27%
EEF1A1	c.22_23delAT	p.Ile8fs	frameshift variant	0%	24%	27%	26%
HGC6.3	c.451_452delCAinsACAC	p.Gln151fs	frameshift variant&missense variant	0%	14%	11%	12%
MTCH2	c.807_808delGTinsAG	p.TrpCys269*	stop gained	0%	25%	23%	22%
TRIM64B	c.377delG	p.Ser126fs	frameshift variant	0%	20%	11%	18%
C12orf49	c.375_385delCAGCGCCTATG	p.Cys125fs	frameshift variant	0%	19%	14%	21%
TAOK3	c.2163dupA	p.Gln722fs	frameshift variant	0%	53%	55%	48%
AKT1	c.430C>G	p.Arg144Gly	structural interaction variant	0%	30%	32%	29%
IKZF3	c.1055C>G	p.Ser352*	stop gained	0%	45%	42%	46%
RALBP1	c.1063C>T	p.Arg355*	stop gained	0%	11%	10%	11%
ERCC1	c.495C>G	p.Ala165Ala	structural interaction variant	0%	53%	44%	47%
FAM227A	c.372+1G>C	n/a	splice donor variant&intron variant	0%	50%	55%	63%
DDX3X	c.1181G>T	p.Arg394Leu	structural interaction variant	0%	60%	49%	52%
RBMX	c.902_903delCGinsTGCC	p.Pro301fs	frameshift variant&synonymous variant	0%	14%	12%	13%

(**H**)

Code	Sex	Age (Y)	subtype	IPSS-R	Therapy (1)	Additional drug	Cycles
P3527	F	71	CMML2	NA	Azacytidine	none	12
P3106	M	74	EB1	Very High	Azacytidine	none	24
P5894	M	76	EB1	Intermediate	Azacytidine	Pevonedistat	23
P5511	M	73	EB1	Intermediate	Azacytidine	Pevonedistat	6

Code	Response at cycle 4 or 8	Therapy (2)	Cycles
P3527	CR	LD-AraC	1
P3106	CR	Guadecitabine	1
P5894	SD-HI	LD-AraC	2
P5511	SD	Rigosertib	6

(**I**)

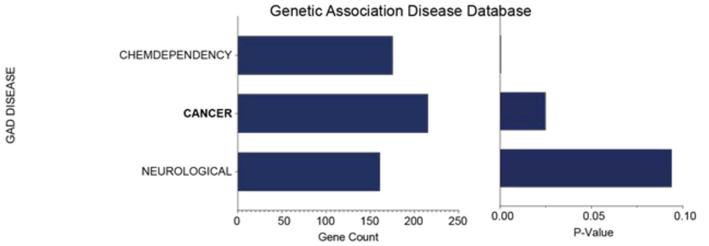

Annotation of AZA-R DNA variants showed significant involvement of AZA-R variants in several **GAD_disease** (Genetic Association Disease Database) categories such as cancer (X axes represent gene count and p-value).

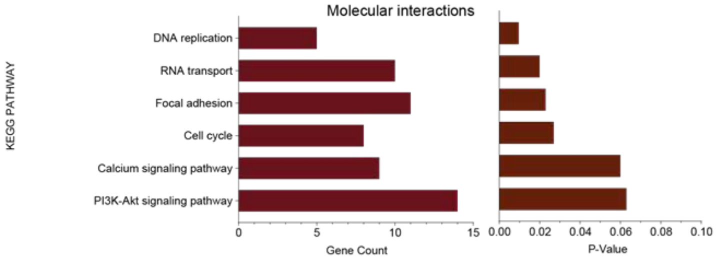

KEGG tool showed involvement in DNA replication, cell cycle and PI3K-AKT signaling pathway (X axes represent gene count and p-value).

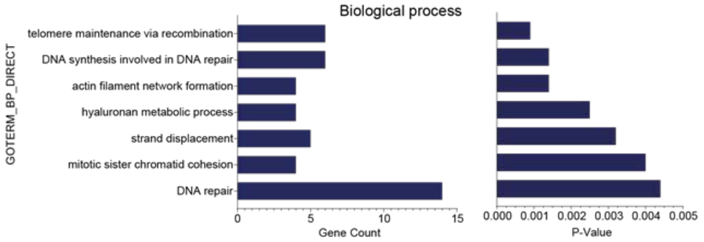

Analysis of the gene ontology in terms of biological process (**GOTERM_BP_DIRECT**) revealed the involvement in telomere length maintenance, chromatid cohesion, DNA repair and base excision repair or regulation of transcription. (X axes represent gene count and p-value).

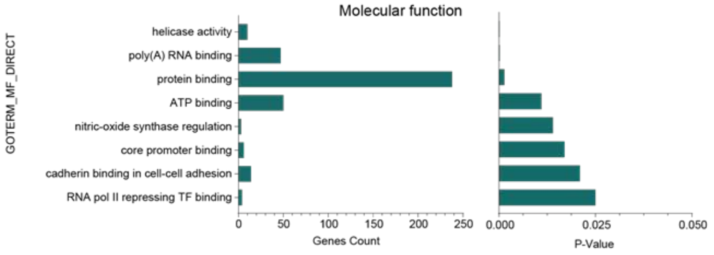

Molecular function analysis of AZA-R variants (**GOTERM_MF_DIRECT**) identified involvement in helicase activity, poly(A) RNA binding, protein binding, and ATP binding (X axes represent gene count and p-value).

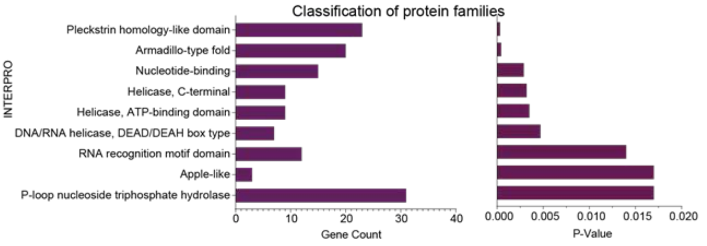

Analysis of AZA-R variants in terms of protein domains by **INTERPRO** showed significant involvement of Pleckstrin homology-like domain, Armadillo-like component, or RNA binding (X axes represent gene count and p-value).

(J)

WES variants (combined)	Categories (gene count)	p value	Genes
GAD_disease	cancer (96)	2E-02	AKT1, BRCA2, ERCC, FANCD2, FANCI, IKZF3, SMARCA2, WT1, RUNX1
UP_KEYWORDS	alternative splicing (304)	7E-13	BCOR, POLD3, SMARCA2, CDC7, CHD7, HDAC2, HDAC6, STAG2
	protein phosphorylation (253)	1E-12	DDX3X, REST, ACVR1, EVI5, EGFR, JMJD1C, MKI67, MAPK6, NOC1, PRMT6, SMC3
KEGG pathway	DNA replication (5)	1E-02	POLD3, DNA2, PRIM1, PRIM2
	cell cycle (8)	3E-02	MAD2L2, CDC7, HDAC2, ORC2, STAG2, SMC3
	PI3K signaling (14)	5E-02	AKT1, EGFR, HGF, PIK3R3, PTK2
GOTERM_BP	telomere length maintenance (6)	9E-04	BRCA2, POLD3, DNA2, WRN, PRIM1, PRIM2
	chromatid cohesion (4)	4E-03	MAU2, NIPBL, PDS5B, SMC3
	DNA & base excision repair (20)	4E-03	DNA2, RAD51AP1, RBBP8, WRN, FANCD2, FANCI, TAOK3, SMC3
	regulation of transcription (20)	5E-02	BCL10, SP100, ANKRD49, EGR1, HDAC2, IRF6, RUNX1, SKAP1, ZFHX3, ASXL1
GOTERM_MF	helicase activity (10)	2E-04	SMARCA2, CHD7, DNA2, DDX3X, DDX60L, DHX36, DDX50, DDX60
	poly(A) RNA binding (47)	2E-04	RBM25, RBM27, TSR1, PABPC1, PARP12, TCERG1, SRFBP1
	protein binding (238)	1E-03	AKT1, ERCC1, BRCA2, FANCD2, CHD7, EGFR, IKZF3, CDC7, MAP3K7
	ATP binding (50)	1E-02	DDX37, DDX60L, SMARCA2, CHD7, PDS5B, SMC3
BIOKARTA	healing and ATRII pathways (8)	4E-02	AKT1, PTK2B, PTK, EGFR, MEF2D
INTERPRO	Pleckstrin homology-like domain (23)	3E-04	AKT1, PLCB3, PTK2B, PTK, SKAP1, TRIO
	Armadillo-like component (20)	4E-04	PDS5B, BHLHB9, TERB1, USP24, USP9Y, ULK4
	Nucleotide binding (15)	3E-03	DDX50, RBM25, RBM27, RBMX2, RNPC3, UPF3B, CSTF2T, PABPC1, PABPC3, SRRT
	Helicase, ATP-binding domain (25)	4E-03	DDX3X, DDX60L, DHX36, DDX50, DDX60, WRN, CHD7, SMARCA2,
BIOGRID	SOCS6 (4)	3E-03	APPL1, CLNK
	CBL (11)	3E-03	EGFR, FRS2, PIK3R3, PTK2B, PTK2, ARHGEF6

WES variants (OCI-M2 AZA-R unique)	Categories (gene count)	p value	Genes
GAD_disease	Tobacco Use Disorder (84)	7E-05	FANCD2, SMARCA2, JMJD1C, WT1, NOS1, GRB14
UP_KEYWORDS	alternative splicing (228)	1E-11	FANCI, ERCC1, IKZF3, RAVER1, PRMT6, CEP76, CDC7, CARD19, SUN3, DNA2
	protein phosphorylation (181)	2E-08	AKT1, BRCA2, DDX3X, DHX36, DDX50, ARHGAP5, SMYD2, CHD7, EGFR, PIP4K2C, PI4KB
KEGG pathway	PI3K signaling (11)	3E-02	INPP4A, MTMR4, PI4KB, PIP4K2C, PIK3R3
	Cancer (5)	2E-02	AKT1, ARHGAP5, EGFR, HGF, RELN
	Fanconi anemia (4)	3E-02	BRCA2, ERCC1, FANCD2, FANCI
GOTERM_BP	regulation of transcription (43)	4E-03	IKZF3, SMARCA2, CHD7, HDAC2, WT1, DDX3X
	DNA repair (11)	8E-03	ERCC1, FANCD2, RAD51AP1, RBBP8, PDS5B
	cell cycle (9)	4E-03	MAPK6, EVI5, CYLD, APPL1
	telomere maintenance (5)	2E-03	BRCA2, DNA2, PRIM1, PRIM2, WRN
GOTERM_MF	helicase activity (9)	1E-04	DDX3X, DDX60L, DHX36, DDX50, SMARCA2, CHD7
	poly(A) RNA binding (33)	2E-04	RBM25, RBM27, TSR1, PABPC1, PARP12, TCERG1, SRFBP1
	protein binding (181)	1E-03	AKT1, IKZF3, HBP1, RBBP8, REST, ARHGEF6, ARHGAP5, SMYD2, ACVR1
	ATP binding (38)	1E-02	DDX36, DDX60L, SMARCA2, CHD7, CDC7, EGFR, MAP3K7, MAPK6
INTERPRO	Pleckstrin homology-like domain (16)	5E-03	AKT1, PTK2B, SKAP1, TRIO, GRB14
	Armadillo-like component (13)	1E-02	ULK4, FRYL, USP24, PDS5B
	Nucleotide binding (11)	1E-02	DDX50, RBM25, RBMX2, RNPC3, SRRT
	Helicase, ATP-binding domain (22)	2E-03	DDX3X, DDX60L, DHX36, DDX50, DDX60, WRN, CHD7, SMARCA2,
BIOGRID	SOCS6 (4)	3E-03	APPL1, CLNK, EGFR, PIK3R3
	Ser/thre kinase (5)	3E-03	IKZF3, EGFR, GRB14

WES variants (Pts AZA-R unique)	Categories (gene count)	p value	Genes
GAD_disease	Tobacco Use Disorder (78)	2E-03	CTBP2, ETV6, CERC2, WRN, GRB14, MYH7, RUNX1, PRKCD, FOXJ3
UP_KEYWORDS	alternative splicing (224)	1E-06	ETV5, DDX1, DDX17, BCOR, PRDM15, RBM26, RAD51AP1
	protein phosphorylation (183)	2E-06	ATR, CTBP2, MYSM1, RANBP2, SMAD4, AK2, CDC25A, CUL5, XPO5
KEGG pathway	cell cycle (9)	2E-03	ATR, E2F1, SMAD4, CDC25A, CDC7, CHEK2, HDAC2, PLK1, PRKDC
	leukemia (5)	4E-02	CTBP2, RUNX1
GOTERM_BP	regulation of transcription (29)	1E-02	ETV5, ETV6, HDAC2, RUNX1, CTNNB1, EGFR
	DNA repair (10)	3E-02	POLA1, WRN, UVRAG, RAD51AP1, RECQL
	cell cycle (14)	2E-02	MKI67, PLK1, CDC25A, SMAD4, KIF2C, REG3A
GOTERM_MF	protein binding (173)	4E-02	BCOR, ATR, CTBP2, SMAD4, CDK12, HHIP, MKI67, NOD2, RUNX1
	ATP binding (45)	6E-04	CDC7, MARK2, PLK1, PRKCD, CHEK2, AK2
	poly(A) RNA binding (37)	5E-04	DDX1, DDX17, DHX36, DDX50, H1FX, ABT1, NOL7
	RNA binding (17)	4E-02	XPO5, PABPC1, PABPC3, RPL21, AHCYL1, RBM26, DIS3
BIOKARTA	cell cycle (9)	3E-03	CDC25A, CHEK2, PLK1, PRKDC
INTERPRO	Proetin kinase domain (17)	3E-02	CDC7, MARK2, TRIO, OLK4, TNK2, PRKDC
	Armadillo-like component (13)	2E-02	ATR, CTNNB1, FHOD1, XPO5
	Helicase, ATP-binding domain (6)	4E-02	DDX1, DDX17, DHX36, DDX50, RECQL, WRN
BIOGRID	TP53 (19)	4E-03	RAD23A, AGO1, CHEK2, PLK1, PRKCD
	CREBBP (9)	3E-02	CTNNB1, RUNX1, TDG, WRN

(**K**)

Figure S1: Validation and Mutation screen of AZA-R model & MDS patients. (**A**) IC50AZA of the AZA-R MDS/AML cell lines used (OCI-M2, MOLM13, SKM1). (**B**) Analysis of cell proliferation of AZA-S vs four independent AZA-R subclones. (**C**) bone marrow cytology of OCI-M2 AZA-R cells derived from CDX mice. Cluster of myeloblasts is indicated by an arrow; (**D**) AZA-S and AZA-R cells were transplanted into NSGS mice and either treated with AZA (AZA-S/AZA-R TREATED) or with vehicle control (AZA-S/AZA-R CTRL). Luciferase signal of the tumor has been monitored each week (W). Therapy of 150 μg AZA/mouse was applied 3 times a week indicated by yellow arrow. Evaluation of luciferase signal in control vs treated mice bearing AZA-S or AZA-R tumors. Unique mouse ID is shown in green or orange. (**E**) Using 33-gene MDS panel we identified variants with high impact in 6 genes previously coupled with MDS pathogenesis. Coding DNA sequence (CDS) mutation and aminoacid (AA) substitution is shown for each gene. Variant alelle frequency VAF (%) is shown for the different clones on the right. (**F**) M-FISH summary of cytogenetic aberrations in AZA-S and AZA-R cells. Below are representative karyotypes from AZA-S, AZA-R prior and after extraction from CDX tumors. Note that some differences observed in the AZA-R CDX cells were not seen consistently within the clone, e.g., trisomy of chr3, chr14. (**G**) WES gene lists. Left panel: using WES we identified high-impact variants that were shared (N=188) among AZA-S and AZA-R subclones (variants with highest VAF are shown, full data set was deposited), right panel: a set of unique (N=349) AZA-R variants (variants with highest VAF are shown, full data set was deposited). In red: genes implicated in AZA resistance. (**H**) Unique variants of AZA-R. WES-identified 19 unique AZA-R variants that were identical across AZA-R clones (but not in AZA-S, green) and are

highly suspected to represent early transformation events for the evolution of the AZA-R phenotype. VAF (%) is shown for the different clones on the right. (**I**) MDS patients (N=4, P3527, P3106, P5894, P5511): P3527 (female, age of 71) was diagnosed with MDS-related CMML2, received 12 cycles of AZA (5+2+2, 75 mg/m2), and responded by complete remission. Upon loss of the response the patient received low dose ARA-C (10 days of 2x20mg s.c./28day cycle) but died as a result of progression-associated infection. Patient P3106 (male, age of 74) was MDS-EB1 (excess of blast 1), Very High Revised International Prognostic Index Score (IPSS-R) [17] Risk, received 24 cycles of AZA therapy, achieved complete remission (CR), and upon loss of response he received Guadecitabine (which is a next-generation hypomethylating agent whose active metabolite decitabine has a longer in-vivo exposure time) but died shortly after disease progression. Patient P5894 (male, age of 76) was MDS-EB1, Intermediate IPSS-R Risk, received AZA/Pevonedistat combination, achieved stable disease with hematologic improvement (SD-HI) after 4 cycles, eventually progressed to MDS/AML after 23 cycles, died after 2 cycles of Low-Dose AraC due to progression. Patient P5511 with MDS-EB1, intermediate IPSS-R risk, received AZA/Pevonedistat combination, progressed after 6 cycles and therefore therapy was changed to rigosertib, but patient died due to progression. (**J**) WES analysis of AZA-R cells. From top to bottom: GAD disease analysis, KEGG pathway - molecular interactions, GOTERM Biological process, GOTERM Molecular function, INTERPRO protein domain involvement. (**K**) WES analysis of AZA-R cells. Table listing the involved genes and pathways from GAD disease analysis, KEGG pathway - molecular interactions, GOTERM Biological process, GOTERM Molecular function, and INTERPRO protein domain involvement. Upper panel: combined analysis, middle panel: annotation of AZA-R variants from OCI-M2 cells, lower panel: annotation of AZA-R variants from AZA-resistant MDS patients.

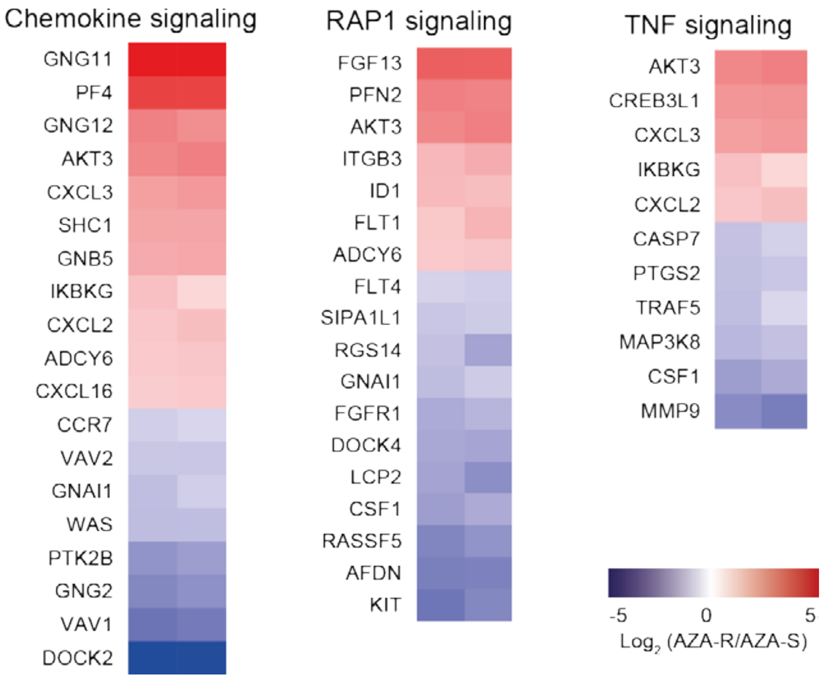

(**A**)

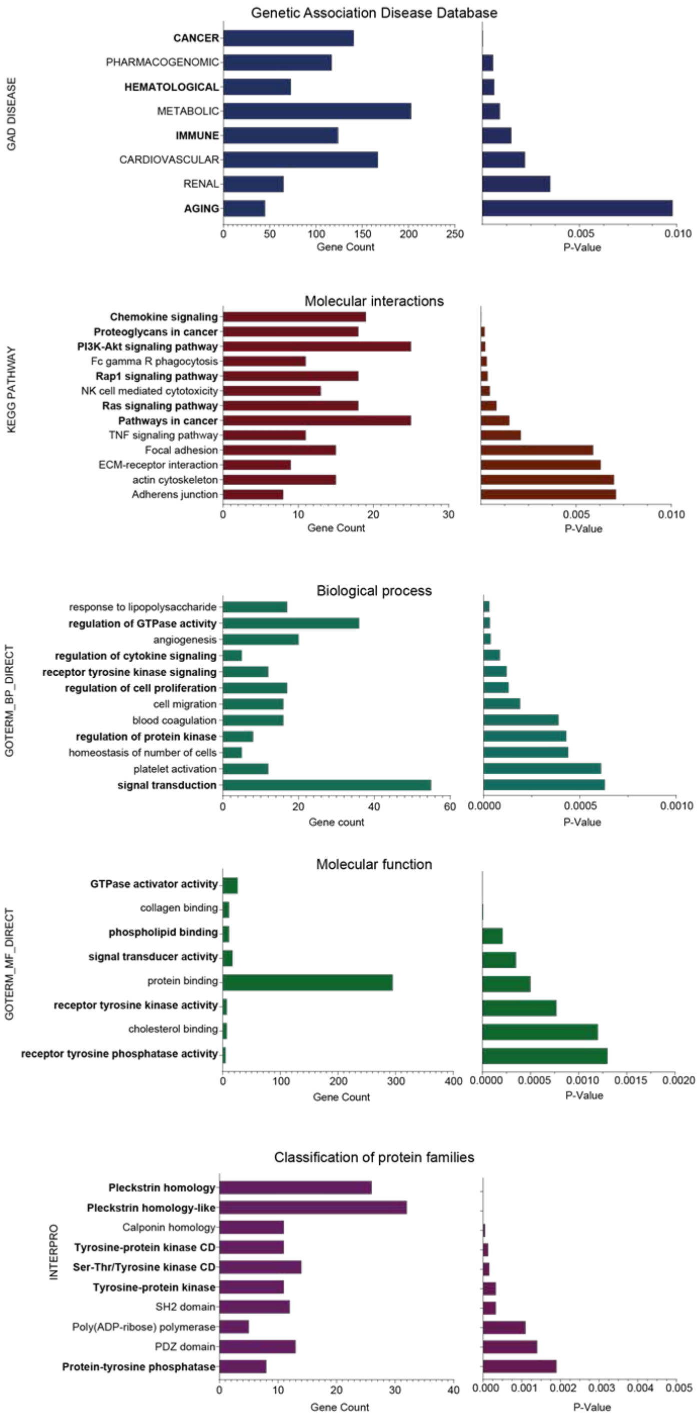

Annotation of differentially expressed mRNAs in AZA-R identified several **GAD_disease** categories, with cancer being the most significant, pharmacogenomics, hematology, and metabolism (X axes represent gene count and p-value).

Analysis of signaling pathways by **KEGG** revealed involvement of the differentially expressed AZA-R mRNAs in chemokine signaling pathway, the PI3K-AKT signaling pathway, and the RAS signaling pathway (X axes represent gene count and p-value).

Analyses of biological processes (**GOTERM_BP_DIRECT**) in the differentially expressed AZA-R mRNA profile revealed cell proliferation and positive regulation of GTPase activity (X axes represent gene count and p-value).

Molecular function analysis (**GOTERM_MF_DIRECT**) of AZA-R DE mRNA profile identified the most significant being the GTPase activity (X axes represent gene count and-p value).

Analysis of DE AZA-R mRNAs using **INTERPRO** indicated involvement of the Pleckstrin homology-like domain and the Pleckstrin homology domain among the most significant ones (X axes represent gene count and p-value).

(**B**)

DE mRNAs	Categories (mRNA count)	p value	Genes
GAD_disease	cancer (141)	3E-05	AKT3, IKZF1, IKZF2, ERG, KIT, SMAD3, CCND1, CDKN2A, CASP4, CASP7
	hematology (117)	6E-04	IKZF1, IKZF3, ERG, SATB1, SMARCA2, RIN3, MAP3K8, GSTM1, CDKN2A, CDCA7, CDH2
	metabolism (203)	9E-04	KIT, SMAD6, SMAD3, WNT5B, ERBB3, FGFR1, FLT1, HOXA10, ID1, ID2, FOXB1, IRAK3, PTK2B
UP_KEYWORDS	protein phosphorylation (290)	6E-10	AKT3, DDX25, EHD2. EHD3, SMARCA1, SMARCA2, TAL1, FLT1, FLT4, PARP10, PTPN6, ABCA1
	alternative splicing (335)	3E-06	ABCA3, POU2F2, RBPMS2, SATB1, SIMC1, TEAD4, CDC14B, CSF2RBENG, FZD3, PARP14, PARP3
KEGG pathway	chemokine signaling pathway (19)	5E-03	CXCL2, CXCL3, CXCL7, CXCL16, AKT3, PTK2B, VAV1, VAV2
	PI3K signaling (25)	2E-04	AKT3, CCND1, CSF1
	RAS signaling pathway (18)	2E-02	CSF1, FGF13, SYK, FLT1, FLT4, FGFR1, KIT, AKT3
GOTERM_BP	cell proliferation (17)	5E-02	BMX, KIT, S100A11, CDCA7, HOXD13, PTK2B, TAL1
	regulation of GTPase activity (36)	3E-05	DLC1, GDI1, RIN3, RASGEF1B, SHC1, AGAP1-3, GNB5, RAP1GAP2
GOTERM_MF	GTPase activity (26)	7E-07	RAP1GAP2, RIN3, RINL, RGS14, RGS20, SIPA1L1, SYDE1
	signal transducer activity (17)	3E-04	BMX, ERG, GNA15, GNAI1, GNB5, GNG11, GNG12, PLCL1, PTK2B
	phospholipid binding (11)	2E-04	ABCA1, AGAP1, SGIP1, SHC1, STAP1
INTERPRO	Pleckstrin homology-like domain (32)	2E-04	SHC1, STAP1, PLEKHA4, PLEKHA6, PLEKHO1, PLEKHG4, PTPN13, VAV1, VAV2
	Pleckstrin homology domain (26)	1E-06	AKT3, BMX, CDH2, AGAP1, ASAP2, ASAP3, ARHGEF6
BIOGRID	CBL (17)	1E-05	KIT, SHC1, CSF1, SMAD3, FLT1, SYK, VAV1, VAV2

(**C**)

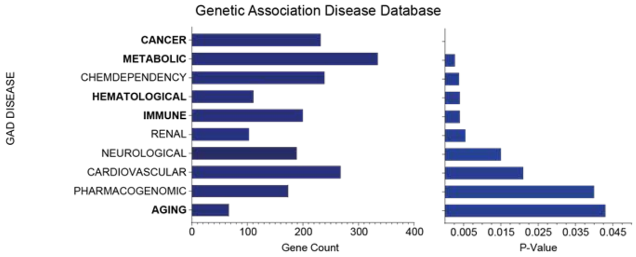

Annotation of differentially expressed DNA variants and mRNAs in AZA-R identified several **GAD_disease** categories, with cancer being the most significant, followed by metabolism and hematology (X axes represent gene count and p-value).

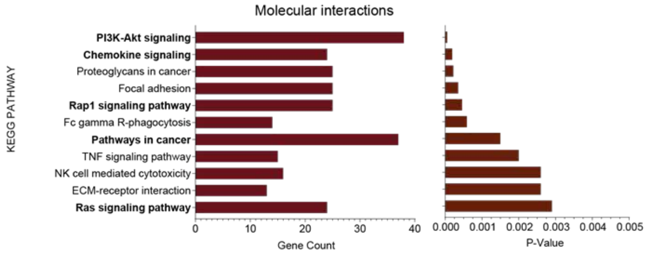

Analysis of signaling pathways by **KEGG** revealed involvement of the DNA variants and differentially expressed AZA-R mRNAs in the PI3K-AKT pathway and chemokine signaling, and the RAS signaling pathway or cancer (X axes represent gene count and p-value).

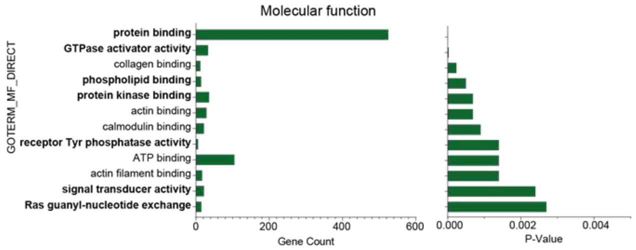

Analyses of biological processes (**GOTERM_BP_DIRECT**) in DNA variants and the differentially expressed AZA-R mRNA profile revealed protein binding, GTPase activity, phospholipid binding, protein kinase binding among the most significant (X axes represent gene count and p-value).

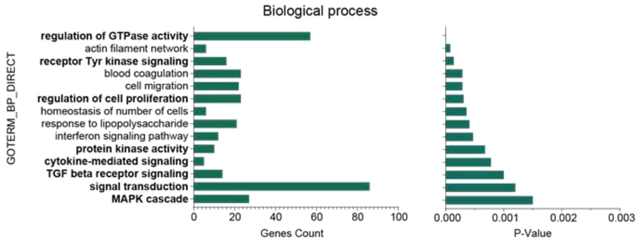

Molecular function analyses (**GOTERM_MF_DIRECT**) of AZA-R DNA variants and DE mRNA profile identified the most significant being the GTPase activity, Tyr kinase signaling, cell proliferation or cytokine mediated signaling among the most significant (X axes represent gene count and p-value).

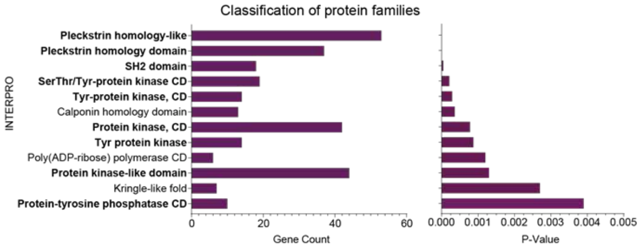

Analysis of DNA variants and DE AZA-R mRNAs using **INTERPRO** indicated involvement of the Pleckstrin homology-like domain and the Pleckstrin homology domain, SH2 domain, protein kinase domain and protein phosphatase among the most significant ones (X axes represent gene count and p-value).

(**D**)

Figure S2: RNAseq and DAVID annotations of AZA-R profiles. (**A**) Transcriptomic analysis of AZA-R vs AZA-S cells. Heatmaps – showing mRNA expression log2 (AZA-R/AZA-S) in two replicates of KEGG-enriched pathways; note that some mRNAs are involved in more than one (see Results, *section* 3.3.). (**B**) RNAseq data analysis of AZA-R cells. From top to bottom: GAD disease analysis, KEGG pathway - molecular interactions, GOTERM Biological process, GOTERM Molecular function, INTERPRO protein domain involvement.(**C**) RNAseq analysis of AZA-R cells. Table listing the involved genes and pathways from GAD disease analysis, KEGG pathway - molecular interactions, GOTERM Biological process, GOTERM Molecular function, and INTERPRO protein domain involvement. (**D**) WES and RNAseq combined analysis of AZA-R. From top to bottom: GAD disease analysis, KEGG pathway - molecular interactions, GOTERM Biological process, GOTERM Molecular function, INTERPRO protein domain involvement.

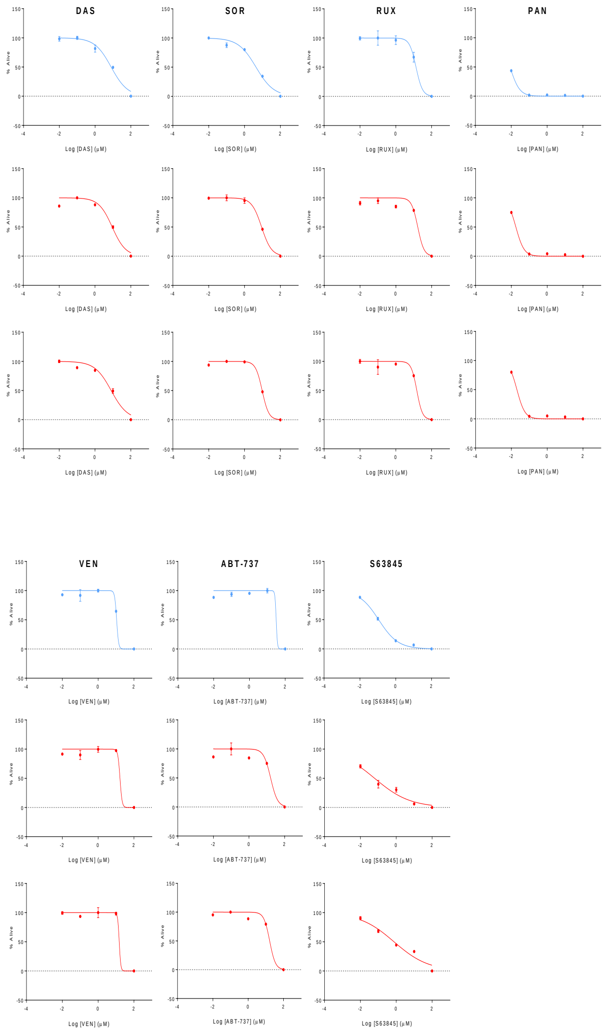

DRUG	IC_{50} (μM)		
	AZA-S	$AZA\text{-}R^{MEAN}$	$AZA\text{-}R^{SD}$
Azacitidine (AZA)	0.063	10.833	1.768
Dasatinib (DAS)	7.894	8.291	0.732
Sorafenib (SOR)	4.383	8.201	1.970
Ruxolitinib (RUX)	13.500	15.507	1.087
Panobinostat (PAN)	0.009	0.020	0.002
Venetoclax (VEN)	10.590	14.410	2.133
ABT-737	31.490	16.503	0.172
S63845	0.112	0.438	0.350
Idelalisib (IDE)	10.200	12.840	3.026
MK2206	0.246	0.257	0.063

(**A**)

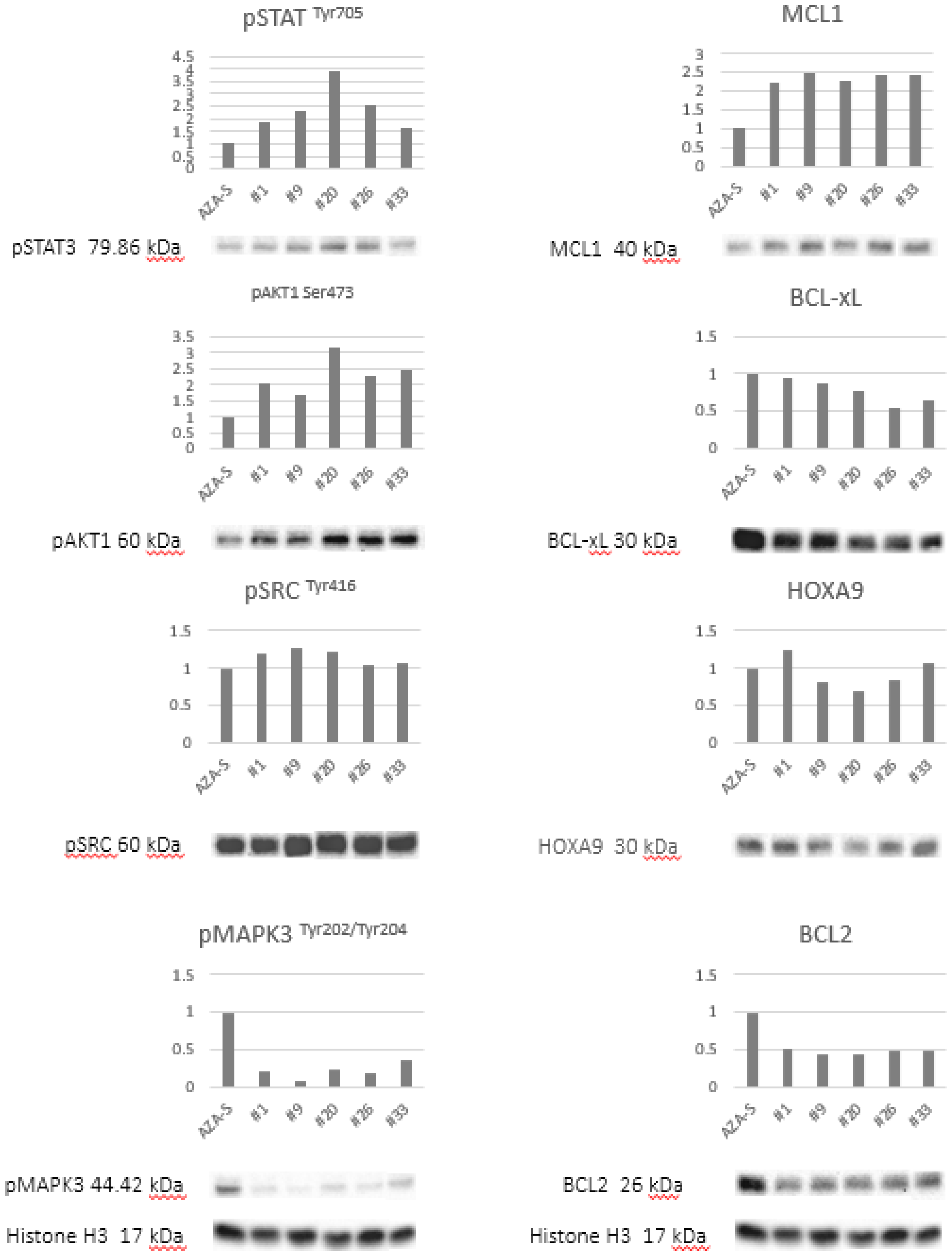
pSTAT Tyr705
MCL1
AZA-S
#1
#9
#20
#26
#33
pSTAT3 79.86 kDa
MCL1 40 kDa
pAKT1 Ser473
BCL-xL
pAKT1 60 kDa
BCL-xL 30 kDa
pSRC Tyr416
HOXA9
pSRC 60 kDa
HOXA9 30 kDa
pMAPK3 Tyr202/Tyr204
BCL2
pMAPK3 44.42 kDa
BCL2 26 kDa
Histone H3 17 kDa
Histone H3 17 kDa

(**B**)

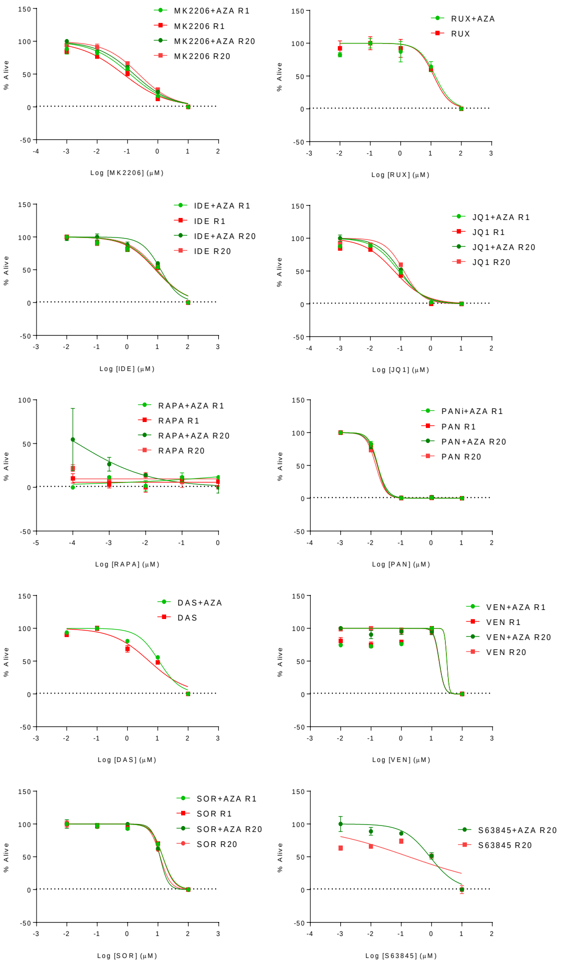

	AZA-R, #1	AZA-R, #20
MK2206	0.07285	0.2347
MK2206+AZA	0.1218	0.1628
p value	0.5405	0.9807
IDE	8.347	9.564
IDE+AZA	8.806	12.5
p value	0.5389	0.6935
RAPA	n/a	n/a
RAPA+AZA	0.0007048	0.0001337
p value	n/a	n/a
DAS	5.215	n/a
DAS+AZA	11.01	n/a
p value	0.7366	n/a
SOR	14.4	11.86
SOR+AZA	14.48	11.7
p value	0.6276	0.7547
RUX	12.39	n/a
RUX+AZA	13.91	n/a
p value	0.9417	n/a
JQ1	0.06047	0.1265
JQ1+AZA	0.08529	0.09781
p value	0.6312	0.9802
PAN	0.01628	0.01446
PAN+AZA	0.0169	0.01651
p value	0.7953	0.8873
VEN	31.42	17.33
VEN+AZA	31.39	17.65
p value	0.4318	0.5793
S63845	n/a	0.07253
S63845+AZA	n/a	0.05138
p value	n/a	0.895

(**C**)

Figure S3: WST1 assay of AZA-R inhibitors in monotherapy and with AZA; Immunoblotting analysis for candidate pathways. (**A**) WST1 assay, AZA-S and AZA-R cells were used. The following inhibitors (DAS, SOR, RUX, PAN, VEN, ABT-737 and S63845) were evaluated in monotherapy using WST1 assay. IC50 for each drug is shown in the table (Idelalisib, JQ1, PAN, and MK2206 data are shown in Fig. 3). (**B**) Western blot analysis of AZA-S and AZA-R subclones. Probed proteins are listed on the top of the figures, Y axis represents density of the bands of gels shown below the histograms, X axis depicts samples of cell lysates. Histone H3 detection represents a loading control. (**C**) WST1 assay, two AZA-R (1, 20) subclones were used. Additive or synergistic effect of the inhibitors (MK2206, IDE, RAPA, DAS, SOR, RUX, JQ1, PAN, VEN, S63845) was tested. Red color indicates monotherapy, green indicates combination with AZA (1**µ**M). IC50 for each drug in two AZA-R subclones is shown in the table.
